# Correlation between Calcitonin Levels and [^18^F]FDG-PET/CT in the Detection of Recurrence in Patients with Sporadic and Hereditary Medullary Thyroid Cancer

**DOI:** 10.5402/2012/375231

**Published:** 2012-05-10

**Authors:** Evangelia Skoura, Ioannis E. Datseris, Phivi Rondogianni, Stylianos Tsagarakis, Marinella Tzanela, Maria Skilakaki, Dimitrios Exarhos, Maria Alevizaki

**Affiliations:** ^1^Nuclear Medicine Department, Evangelismos General Hospital, Ipsilantou 45-47, 10676 Athens, Greece; ^2^Department of Endocrinology, Evangelismos General Hospital, Ipsilantou 45-47, 10676 Athens, Greece; ^3^Department of Endocrinology, Polikliniki General Hospital, Pireos 3, 10552 Athens, Greece; ^4^Department of Radiology and CT Department, Evangelismos General Hospital, Ipsilantou 45-47, 10676 Athens, Greece; ^5^Department of Endocrinology, Alexandra General Hospital, Vassilissis Sofias 80, 11528 Athens, Greece

## Abstract

*Purpose*. Measurement of serum calcitonin is important in the followup of patients with medullary thyroid carcinoma (MTC) and reliably reflects the presence of the disease. This is the largest study so far in bibliography investigating the diagnostic accuracy of combined [^18^F]FDG-PET/CT in patients with MTC and elevated calcitonin levels. *Methods*. Between February 2007 and February 2011, 59 [^18^F]FDG-PET/CT were performed on 51 patients with MTC and elevated calcitonin levels for localization of recurrent disease. Conventional morphologic imaging methods were negative or showed equivocal findings. *Results*. Among the 59 [^18^F]FDG-PET/CT, 29 were positive (26 had true-positive and 3 false-positive findings) and 30 negative. The overall per-patient sensitivity of [^18^F]FDG-PET/CT was 44.1%. Using as cut-off point the calcitonin value of 1000 pg/ml, in patients with calcitonin exceeding this value, sensitivity raised to 86.7%. The overall sensitivity of [^18^F]FDG-PET/CT was lower (23%) in patients with MEN IIA syndrome. *Conclusion*. The findings of this paper show that [^18^F]FDG-PET/CT is valuable for the detection of recurrence in patients with highly elevated calcitonin levels, >1000 pg/mL, but in patients with lower calcitonin levels, its contribution is questionable. Also, there is evidence that the sensitivity of [^18^F]FDG-PET/CT is lower in patients with MTC as part of MEN IIA syndrome.

## 1. Introduction

Inherited and sporadic medullary thyroid cancer (MTC) is an uncommon and challenging malignancy. Its low incidence has limited both widespread clinical expertise and definitive randomized clinical trials [1]. It originates from the parafollicular calcitonin-secreting cells of the thyroid, explaining the key role of calcitonin as a specific and sensitive marker of this cancer [2]. MTC may occur in sporadic (75% of cases) or hereditary (25% of cases) forms that include multiple endocrine neoplasia (MEN) types IIA and IIB and isolated familial MTC [3, 4].

When no distant metastasis is present, the curative treatment for MTC is total thyroidectomy and lymph node dissection [1, 5, 6]. Measurements of the serum calcitonin and CEA are important in the followup of patients with MTC and reliably reflect the presence and volume of disease in the vast majority of them [1, 7]. These tumor markers typically require several months after surgery to achieve their nadir [1, 7]. At 2-3 months after surgery, a basal calcitonin level is undetectable in 60%–90% of patients without initial lymph node involvement and in less than 20% of patients with lymph node metastases. If calcitonin is then undetectable, a pentagastrin stimulation test may be performed to exclude any residual disease [1, 8]. When both the basal and the stimulated serum calcitonin are undetectable, the patient is in complete biochemical remission and has about a 3% chance of biochemical recurrent disease during followup [9]. It is reported that biochemical cure predicted a survival rate of 97.7% at 10 years [10].

Within the past decade the prognosis has improved mainly because of earlier diagnosis and improvement in surgical procedures [10, 11]. Nevertheless, more than 50% of thyroidectomized patients are not cured after surgery, as persistent elevation of basal serum calcitonin levels, which implies residual tumor, is frequently observed after primary surgery [5, 10]. In these cases, additional surgery is a treatment option to achieve biochemical remission only when specific, resectable lesions are evident by imaging studies [5]. Recent ongoing trials with some novel compounds, directed against angiogenesis and molecular targets in tumor cells, have been shown promising [1]. However, the most important prognostic factor in patients with recurrent MTC remains the early diagnosis that facilitates early surgical intervention before metastatic spread outside the thyroid bed [12]. Until now, there is no single sensitive diagnostic imaging method to reveal all MTC recurrence or metastasis. Conventional morphologic imaging methods (U/S, CT, MRI) and several methods of nuclear medicine have been used for this purpose with variable accuracy [13]. [^18^F]FDG-PET has an established role in the restaging of various cancers [14]. However, controversy exists regarding its ability to assess reliably recurrent or persistent MTC.

In this paper we studied the diagnostic accuracy of [^18^F]FDG-PET/CT, in patients with MTC and elevated calcitonin levels. Although there are published studies dealing with the same subject, this study constitutes the one with the largest cohort of patients with MTC examined with combined [^18^F]FDG-PET/CT scans.

## 2. Materials and Methods

This is a prospective study of [^18^F]FDG-PET/CT scans performed for localization of recurrent disease on patients with histologically proven MTC, elevated calcitonin levels, and negative or equivocal conventional imaging findings. From February 2007 to February 2011, 59 [^18^F]FDG-PET/CT scans were performed on 51 patients with MTC. The group included 15 men and 36 women, 12–79 years old (mean:  53 ± 15.17  years). 18 patients had hereditary MTC (7 familial MTC and 11 as part of MEN IIA syndrome) and 33 sporadic MTC. All patients underwent total thyroidectomy as initial treatment. 36 among them had neck lymph node dissection as well, while 5 patients had mediastinal node dissection. In all 11 patients, with MEN IIA-related MTC, adrenalectomy had been performed for pheochromocytoma, and in 4 of them subtotal parathyroidectomy had been performed for glandular parathyroid hyperplasia. Median followup after initial surgery until performing [^18^F]FDG-PET/CT was 8.4 years (range, 4 months–38 years). Characteristics of the study population are listed in Table 1.

All patients were asymptomatic and all had elevated serum calcitonin levels (19.3–21000 pg/mL, normal value: <10 pg/mL). 22 patients also had elevated CEA levels (7.2–130 ng/mL, normal value: <5 ng/mL).

A second [^18^F]FDG-PET/CT scan was performed in 8 patients when there was an increase by at least 20% in calcitonin levels and the prior scan was negative or when the prior scan was positive, and although the patient had undergone surgical resection of the MTC recurrence, the calcitonin level still had not normalized.

As previous studies have shown, [^18^F]FDG-PET/CT scan has the greatest sensitivity in patients with calcitonin levels of at least 1,000 pg/mL, and there is no significant difference in sensitivities in cases with any value below this value [15, 16]. We calculated the sensitivity in patients with calcitonin levels exceeding 1000 pg/mL (15 cases).

All patients underwent additional examinations with at least one other imaging method, depending on the local preferences of the different centres, the previous 4 weeks (5–28days), that had shown negative or equivocal findings. They were either negative or showed equivocal findings: 25 patients underwent standard dose CT scan, 28 MRI, 32 U/S of the neck, 14 ^111^In-pentetreotide, 13 ^123/131^I MIBG, 9 bone scan and 1 ^99 m^Tc-(V)DMSA.

The study was approved by the institutional ethical committee, and all patients gave written informed consent.

### 2.1. Image Acquisition

#### 2.1.1. PET/CT

 A standard whole-body protocol and an additional dedicated neck [^18^F]FDG-PET/CT protocol were used in all patients. The patients were asked to fast for 6 hours before the study. The serum glucose concentration, before the injection of [^18^F]FDG, was less than 150 mg/dL. The image acquisition started about 60 min after the intravenous administration of a dose of 5 MBq/Kgr [^18^F]Fluorodeoxyglucose—[^18^F]FDG. All acquisitions were performed by using an integrated PET/CT scanner (Discovery ST; GE Medical Systems). The average total PET/CT examination time was 35 minutes.

Whole-body image from the mid femur to the base of the brain was obtained, divided usually in 6 bed positions. The PET emission images were acquired for a 4-minute acquisition period at each bed position. Imaging system enabled the simultaneous acquisition of 47 transverse PET images per field of view with intersection spacing of 3.27 mm, for a total transverse field of view of 15.7 cm. PET resolution is approximately 6.1 mm full width at half maximum near the centre of the field of view. The PET/CT system also includes a 4-detector row helical CT scanner (140 kV and 80 mA). The CT images were used not only for image fusion but also for generation of the attenuation map for attenuation correction. PET scan was acquired in the two-dimensional mode (2D). The field of view and pixel size of the reconstructed images were 50 cm and 3.91 mm, respectively, with a matrix size of 128 × 128. The reconstruction method used was filtered back projection with Hanning filter.

In neck dedicated protocol, the acquisition started immediately after the whole-body scan, and the field of view and pixel size of the reconstructed image were 30 cm and 2.34 mm respectively, with a matrix size of 128 × 128. The reconstruction method used was filtered back projection with Hanning filter.

PET/CT scans were interpreted visually by both a nuclear medicine physician and a radiologist. The evaluation included calculation of the overall per-patient sensitivity of [^18^F]FDG-PET/CT as well as calculation of this parameter after division of the scans performed into two groups according to calcitonin levels.

### 2.2. Interpretation

Standard whole-body PET/CT images were reviewed on the Xeleris workstation in transverse, coronal, and sagittal planes, along with maximum intensity projection images. For visual analysis, ^18^F-FDG PET uptake was considered abnormal if located outside the normal anatomic structures or if having intensity greater to the background blood-pool activity or adjacent normal tissue. In addition, standardized uptake value (SUV) of the lesions was measured on the standard whole-body PET/CT in a semi-quantitative factor. SUV was calculated using the following formula:

(1) $$\text{SUV}=\frac{Cdc}{di/w},$$

where Cdc is the decay-corrected tracer tissue concentration (in becquerels per gram), di is the injected dose (in becquerels), and w is the patient's body weight (in grams). The maximum SUV (SUVmax) was recorded for each lesion after applying regions of interest (ROI) in the transaxial attenuation corrected PET slices, around the pixels showing the greatest accumulation of ^18^F-FDG.

### 2.3. Data Analysis

Imaging findings were classified as true-positive for local recurrence or metastasis if confirmed by one of the following criteria: (a) positive histopathology results from biopsies or resections, (b) the presence of a detectable lesion at the corresponding site on follow-up conventional imaging studies, (c) an increase in the lesion size and/or in [^18^F]FDG uptake on [^18^F]FDG-PET/CT follow-up scans. Because all patients presented with elevated calcitonin level, any imaging study not showing a clear abnormality was classified as false negative. The [^18^F]FDG-PET/CT scans that had findings proved to be due to other reasons and not to MTC, were also classified as false negative scans in the calculation of the sensitivity of the method. The reason is that they did not detect the real lesions of MTC recurrence responsible for the elevation of calcitonin level.

## 3. Results

59 [^18^F]FDG-PET/CT scans performed in 51 patients were included in this study. Among these, 8 scans from 8 patients represented follow-up scans. Of these follow-up scans, 6 were performed because there was an increase in calcitonin level by at least 20% with a negative previous [^18^F]FDG-PET/CT. The other 2 were performed in patients with a positive prior scan who had undergone surgical resection of disease, but the calcitonin level did not normalize. The median interval between these scans was 12.38 ± 5.21 months (range, 8–24 months).

There were 29 positive and 30 negative [^18^F]FDG-PET/CT scans for detection of MTC recurrence. Two of the negative (for MTC) scans had findings due to recurrence of pheochromocytoma in two MEN IIA patients (patients no. 6 and 34); two further scans (one negative and one positive for MTC) of one patient had findings attributed to adrenal cortical hyperplasia due to Cushing's syndrome caused by ectopic ACTH production (patient no. 5). The latter was histologically proven as the patient underwent bilateral adrenalectomy.

In 30 [^18^F]FDG-PET/CT scans, no abnormal [^18^F]FDG uptake was identified, and therefore these were classified as false negative because all patients had elevated calcitonin levels. One patient of them (with calcitonin level: 77 pg/mL) underwent surgical resection because of abnormal findings in U/S of the neck, even though the [^18^F]FDG-PET/CT scan was negative, but the biopsy was negative for MTC (patient no. 7).

**Figure 1:**
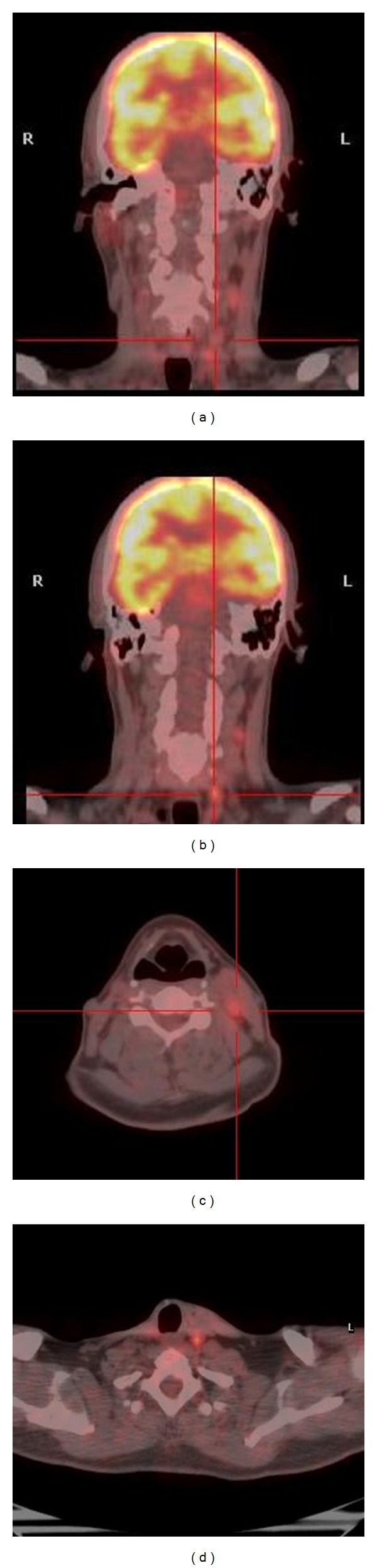
[^18^F]FDG-PET/CT images in a 40-year-old man with known MTC, as part of MEN IIA syndrome and calcitonin levels of 2096 pg/mL, about 9 years after the initial treatment. Images show increased uptake of ^18^FDG in left cervical lymph nodes (SUVmax: 5). The scan was proved true positive as there was histological confirmation.

**Figure 2:**
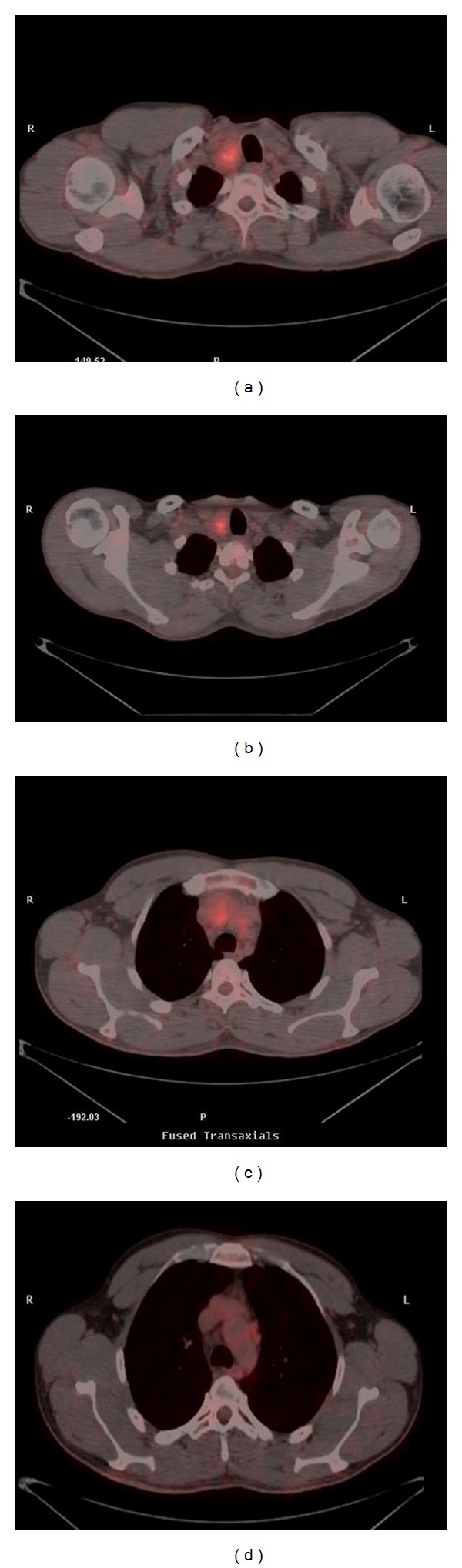
[^18^F]FDG-PET/CT images in a 40-year-old man with known familial MTC and calcitonin level of 6000 pg/mL, about 4 years after the initial treatment. Images show increased uptake of ^18^FDG in the right side of thyroid bed (SUVmax: 4.6) and in prevascular lymph nodes, in the mediastinum. The scan was proved true-positive as there was histological confirmation.

**Figure 3:**
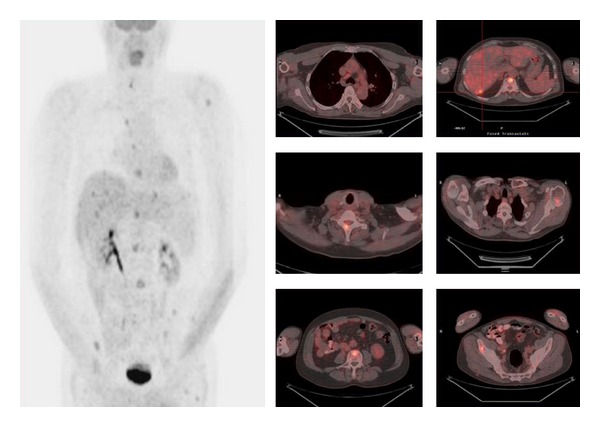
[^18^F]FDG-PET and [^18^F]FDG-PET/CT images in a 59-year-old man with known MTC, as part of MEN IIA syndrome and calcitonin levels of 4800 pg/mL, about 2 years after the initial treatment. Images show increased uptake of ^18^FDG in multiple hepatic lesions (SUVmax: 6), a precarinal lymph node (SUVmax: 2.9) and in multiple bone lesions (SUVmax: 6.1).

Of the 29 positive scans, in 12 surgery was obtained, and there were biopsy results. Nine scans were proved true-positive, with cervical lymph node metastases (patients no. 9, 10B, 26, 28, 37, and 47 (Figure 1)), recurrence in thyroid bed (patients No. 12 and 26), or/and mediastinal lymph node metastases (patients no. 12 and 30 (Figure 2)) of MTC. Three positive scans for cervical lymph node metastases proved to be false-positive, as the biopsy showed only reactive lymphadenitis in these lymph nodes (patients no. 25, 31, and 33). Five of the patients with positive scans could not have resection of the lesions, as there was widespread dissemination of the disease (patients no. 2, 4, 11, 29, 41, and 43 (Figure 3)). The 12 remaining scans deemed true-positive due to the presence of a detectable lesion at the corresponding site on follow-up conventional imaging studies or the increase in lesion size and/or in [^18^F]FDG uptake on [^18^F]FDG-PET follow-up scans. In cooperation with the referral endocrinologists and after all the appropriate clinical and laboratory examinations, other possible pathologies in these patients were ruled out.

In positive [^18^F]FDG-PET/CT scans the lesions were located in the cervical lymph nodes (20), mediastinal lymph nodes (10), thyroid bed (5), liver (3), bones (2), lungs (1), and abdominal lymph nodes (1). In two patients the CT component of PET/CT (low dose CT) showed multiple small pulmonary nodules with no ^18^FDG uptake, probably because of their small size (<1 cm) (patients no. 2 and 4) and in another patient the CT component showed a pulmonary nodule 8 mm with no ^18^FDG uptake (patient 39).

Among the 8 follow-up scans, in the 8 patients that underwent a second scan, six were true-positive for detection of MTC recurrence and two negative.

Overall, [^18^F]FDG-PET/CT scans were positive, indicating the detection of possible MTC lesions, in 49.2% (29/59) of patients with increased serum calcitonin levels and either negative or equivocal conventional imaging. According to the criteria we set for characterization of findings as true positive or false negative, the overall per-patient sensitivity of [^18^F]FDG-PET/CT was 44.1% (26/59) in detecting MTC lesions in patients with increased serum calcitonin levels and either negative or equivocal conventional imaging. After division of the scans performed according to calcitonin levels, in cases with calcitonin level of up to 1000 pg/mL we found a sensitivity of 29.5% (13/44), and in cases with calcitonin level >1000 pg/mL sensitivity was as high as 86.7% (13/15).

In the group with calcitonin level greater than 1000 pg/mL, there were two negative scans for MTC, both from the same patient with MEN IIA syndrome (patient no.6). In his second scan there were findings only due to local recurrence of pheochromocytoma, as already mentioned.

Thirteen [^18^F]FDG-PET/CT scans were performed in 11 patients with MEN IIA syndrome, and 3 of them were positive while 10 were negative (sensitivity 23%). Positive were the [^18^F]FDG-PET/CT scans from 3 patients with very high calcitonin levels 5500, 2096, and 4800 pg/mL, respectively. Therefore, all patients with calcitonin levels <1000 pg/mL had negative [^18^F]FDG-PET/CT scans while only cases with calcitonin levels >1000 pg/mL had positive results (3 of 5 cases). When we excluded these patients with MEN IIA syndrome, then the overall per-patient sensitivity of [^18^F]FDG-PET/CT in detecting MTC lesions increased to 50% and in cases with calcitonin levels >1000 pg/mL increased to 100%.

The calcitonin levels ranged from 48.6 to 21000 pg/mL (average 3347 pg/mL) in patients with true-positive [^18^F]FDG-PET/CT scans and from 19.3 to 1203 pg/mL (average 361 pg/mL) in these with negative studies, while the CEA levels ranged from 1.2 to 130 ng/mL (average 33.71 ng/mL) and from 0.63 to 114.7 mg/mL (average 17.77), respectively. The mean value of SUVmax of all lesions showing [^18^F]FDG uptake in true-positive [^18^F]FDG-PET/CT scans was 3.76 ± 1.29 (range, 2–7) (Table 1). The 3 patients with the false-positive [^18^F]FDG-PET/CT had calcitonin levels of 35 pg/mL, 33.4 pg/mL, and 26.4 pg/mL, and SUVmax 3, 6.7, and 4.4, respectively.

The overall median followup of the patients after [^18^F]FDG-PET/CT was  25 ± 11  months (range, 2–47months).

## 4. Discussion

As MTC secretes calcitonin, it is a highly sensitive and the most specific marker for this tumor [17]. Despite aggressive surgery, there is a significant group of patients who will have persistently elevated calcitonin levels postoperatively [13]. Postsurgically elevated or increasing calcitonin levels strongly suggest the presence of residual or recurrent MTC, and its elevated serum concentration can be observed much earlier than a metastatic focus can be visualized by imaging [18]. It has been estimated that the calcitonin serum level of 1000 pg/mL, which is 100 times the upper normal value limit, indicates on 1 cm^3^ of tumor tissue, although this ratio is variable [19]. Nevertheless, calcitonin estimation is a good measure of tumor volume as the higher the calcitonin level, the greater the chance that the patient has demonstrable distant metastases [18, 20].

Clinical recurrences are often found at an early stage because elevated calcitonin levels lead to their compulsive search, and then disease will usually progress slowly with time. Survival after recurrence may, thus, extend over decades [4]. Indeed, as for any tumor, prognosis is related to both tumor burden and progression rate [19]. Thus, it is particularly important to identify metastases early, employing not only serologic markers such as calcitonin but also imaging techniques as well [14]. Assessment of tumor burden requires a combination of multiple imaging modalities because metastases in the MTC patients often involve multiple organs and tissues and are often multiple in each involved organ, as recently reported on the present series of patients [21]. There is no single sensitive diagnostic imaging method to reveal all MTC recurrences or metastases [13, 17, 22, 23].

PET complements anatomic imaging by adding unique metabolic information to the characterization of malignancy. [^18^F]FDG-PET/CT has the great advantage of combining functional and anatomic imaging at the same time, following image fusion.

 It seems that [^18^F]FDG-PET can play a major role in the followup of patients with postoperative elevated plasma calcitonin, and it leads to selection of patients for secondary surgical intervention [23, 24]. The sensitivity of [^18^F]FDG-PET or [^18^F]FDG-PET/CT for recurrence and residual disease detection per patient is reported to be 47.4%–85% [5, 12–17, 21, 25–29]. [^18^F]FDG-PET also provides additional information in a significant fraction of cases (up to 54%) [14]. Comparing [^18^F]FDG-PET with conventional morphologic imaging methods (U/S, CT, MRI) and functional imaging methods with single-photon emitters in several studies, it can be noted that the [^18^F]FDG PET revealed metastatic lesions in a higher percentage of patients [12, 24–26]. Other studies have suggested that [^18^F]FDG PET imaging is more sensitive in patients with rapidly progressive disease than in patients with slowly rising calcitonin levels [17].

Data from our previous study and that of other studies indicate that [^18^F]FDG-PET or [^18^F]FDG-PET/CT has its greatest utility in patients with calcitonin level greater than 1000 pg/mL [15, 16, 25, 26]. Using an arbitrary cut-off of 1000 pg/mL, the sensitivity for lesion detection in suspected residual, recurrent, or metastatic MTC increased, in two different studies from 62% and 47.4% to 78% and 80%, respectively [15, 16]. These data also suggest that [^18^F]FDG-PET and [^18^F]FDG-PET/CT have limited usefulness in patients with low calcitonin levels (<1000 pg/mL), as the overall sensitivity was only 20%–36.8% [15, 16, 26]. In one study, the sensitivity had no significant difference when the calcitonin levels were below 500 pg/mL or 500–1000 pg/mL [16]. The above results are in accordance with the present study.

This study showed an overall sensitivity of 44.1% for [^18^F]FDG-PET/CT in patients with increased serum calcitonin levels and negative or equivocal conventional imaging. Sensitivity was only 29.5% for patients with calcitonin levels up to 1000 pg/mL. However, [^18^F]FDG-PET/CT detected recurrence or metastasis in 86.7% of patients, when the calcitonin level was greater than 1000 pg/mL.

In fact, the relatively low lesion detection rate in patients with low calcitonin levels is likely a reflection of microscopic disease or a smaller tumor burden. In general, a small lesion size and slow growth rate are known limitations of [^18^F]FDG-PET in several neuroendocrine tumors [30].

An interesting finding of the study was that among the patients with MEN IIA syndrome the sensitivity of [^18^F]FDG-PET/CT for MTC recurrence was significantly lower (23%), and for patients with calcitonin levels <2000 pg/mL this fell to zero (0%). When we excluded the patients with MEN IIA syndrome, then the overall per-patient sensitivity of [^18^F]FDG-PET/CT in detecting MTC lesions increased from 44.1% to 50%, and in the group with calcitonin level greater than 1000 pg/mL the sensitivity increased from 86.7% to 100%. These findings are in accordance with the results of other studies which support that MEN IIA disease induce more indolent MTCs, and as [^18^F]FDG uptake relies on the biological aggressiveness of the tumor, the detection sensitivity of the method is low [16, 31, 32].

In our study, in positive for MTC [^18^F]FDG-PET/CT scans, the mean value of SUVmax of all lesions showing [^18^F]FDG uptake in true-positive [^18^F]FDG-PET/CT scans was 3.76 ± 1.29 (range, 2–7), which is relatively low and may reflect the more indolent nature of many MTC lesions, and it is in agreement with literature data [15, 20, 21].

## 5. Conclusions

It seems that the sensitivity of [^18^F]FDG-PET/CT scan for the detection of MTC recurrence, in patients with elevated calcitonin levels and negative or equivocal conventional imaging findings, is determined by the level of serum calcitonin. The results from this cohort of patients suggest that [^18^F]FDG-PET/CT provides additional information in almost half of all cases (44.1%) detecting occult sites of calcitonin production or confirming equivocal findings of other imaging modalities. However, when the calcitonin levels were greater than 1000 pg/mL, this rate increased to 86.7%.

By inference, [^18^F]FDG-PET/CT appears a valuable tool for the detection of recurrence in patients with highly (>1000 pg/mL) elevated calcitonin levels. In patients with lower calcitonin levels it cannot offer too much. It also seems that the sensitivity of this method is better in patients with sporadic or familial MTC than in those with MTC as part of MEN IIA syndrome. Nevertheless, additional prospective studies are necessary to confirm this conclusion.

## Figures and Tables

**Table 1:** Patients' characteristics and [^18^F]FDG-PET/CT findings.

Patient no.	[^18^F]FDG-PET/CT scan no.	Age (years)/sex	Time from initial surgery to PET/CT (months)	TNM initial staging	Type of MTC	Calcitonin-CEA (pg/mL-ng/mL)	[^18^F]FDG-PET/CT findings (SUVmax)	[^18^F]FDG- PET/CT classification	Biopsy-surgical excision
1	A	32/F	192	T1N1aM0	MENIIA	688–21.16	—	FN	—
	B		216			821-NA	—	FN	—

2		57/F	216	T2N1aM0	Fam	21000-NA	Neck (3), mediastinum (2.8), abdominal (3)	TP	—

3	A	44/F	21	T4aNIbMo	S	809–17.4	—	FN	—
	B		30			1601-NA	Thyroid bed (2)	TP	—

4		71/M	83	T3N1bM0	S	3604–54.2	Neck (2.7)	TP	—

5	A	63/F	4	T3N0M0	S	205–7.2	—	FN	—
	B		13			352-NA	Liver (4.7)	TP	—

6	A	49/M	156	T2N1aM0	MENIIA	1001–39.8	—	FN	—
	B		164			1203-NA	—	FN	—

7		39/F	24	T3N1bM0	S	77–0.63	—	FN	Ex = (−) for MTC

8		43/F	11	T1N1aM0	S	52.2–3.4	—	FN	—

9		40/M	84	T2N1bM0	S	2259–22.7	Neck (4)	TP	Ex = (+) for MTC

10	A	63/M	240	T3N0M0	S	705-NA	Neck (2.5)	TP	—
	B		252			860-NA	Neck (3.7)	TP	Ex = (+) for MTC

11		21/F	60	T3N1bM0	S	1470-NA	Neck (2.1), mediastinum (2.2)	TP	—

12	A	40/M	48	T3N1bM0	Fam	3601–1.3	Thyroid bed (3.9), mediastinum (3.7)	TP	Ex = (+) for MTC
	B		63			6000-NA	Thyroid bed (4.6), mediastinum (4.2)	TP	Ex = (+) for MTC

13		52/F	36	T3N1bM0	S	850–114.7	—	FN	—

14		49/F	84	T1N1bM0	S	936–84.4	—	FN	—

15		35/F	216	T1N0M0	MENIIA	79.1–2.1	—	FN	—

16		46/F	120	T3*Ν*1bM0	MENIIA	230–10.4	—	FN	—

17		56/F	4	T2N1bM0	S	63–12.4	—	FN	—

18		60/F	48	T1NIbMo	S	98.8-NA	Mediastinum (2.5)	TP	—

19		61/M	36	T1N1bM0	S	57–7	—	FN	—

20		68/F	30	T3N1aM0	S	145–7.1	—	FN	—

21		67/M	120	T3N0M0	MENIIA	200–11	—	FN	—

22		70/M	36	T1N1aM0	MENIIA	28.3–1.4	—	FN	—

23		55/F	84	T1N1aM0	S	95.1-NA	—	FN	—

24		53/F	108	T3N1aM0	Fam	10704–72,9	Neck (5.5)	TP	——

25		60/F	9	T2N0M0	S	35-NA	Neck (3)	FP	Ex = (−) for MTC

26	A	47/F	60	T1N1bM0	S	883–34.5	—	FN	—
	B		70			1360–40.2	Thyroid bed (3.2), Neck (3.5)	TP	Ex = (+) for MTC

27		55/F	72	T1N0M0	Fam	100-NA	Neck (2.2)	TP	—

28		28/F	84	T1N1bM0	S	622–13.4	Neck (6.3)	TP	Ex = (+) for MTC

29		66/F	228	T1N1bM0	S	17641-NA	Neck (2.5), mediastinum (4), Bones (5)	TP	—

30	A	73/M	84	T3N1bM0	S	450–130	Mediastinum (4.3)	TP	Ex = (+) for MTC
	B		96			500-NA	Neck (7), mediastinum (3)	TP	—

31		42/F	72	T1N0M0	S	33,4-NA	Neck (6.7)	FP	Ex = (−) for MTC

32		72/M	132	T1N1bM0	S	36.6–4.1	—	FN	—

33		38/F	132	T1N1aM0	Fam	26.4–1	Neck (4.4)	FP	Ex = (−) for MTC

34		43/F	204	T1N0M0	MENIIA	26.2–2.2	—	FN	—

35		64/F	84	T4N1bM0	S	410–7.2	Neck (3.9)	TP	—

36		70/F	456	T1N0M0	S	295–1.2	Neck (5)	TP	—

37		40/M	108	T1N1aM0	MENIIA	2096–22.3	Neck (5)	TP	Ex = (+) for MTC

38		60/F	180	T1N0M0	S	330-NA	—	FN	—

39		79/M	8	T1N0M0	S	137–5	—	FN	—

40		69/F	48	T3N0M0	S	453–10.2	—	FN	—

41		44/F	300	T1N0M0	MENIIA	5500-NA	Thyroid bed (3.5), Neck (2.6), Liver (5)	TP	—

42		62/M	120	T1N1bM0	MENIIA	101–1.9	—	FN	—

43		59/M	21	T1N0M0	MENIIA	4800–67	Liver (6), mediastinum (2.9), bones (6.1)	TP	—

44		45/F	7	T3N1bM0	S	650-NA	Mediastinum (2.7)	TP	—

45		29/F	228	T1N0M0	S	986-NA	—	FN	—

46		42/F	144	T1N1bM0	Fam	893–34.9	—	FN	—

47		52/F	8	T2N0M0	S	291–1.2	Neck (3.5)	TP	Ex = (+) for MTC

48		12/M	7	T3N1aM0	Fam	92.4–4.5	—	FN	—

49		74/F	120	T1N0M0	S	48.6–4.6	Neck (2.4)	TP	—

50		65/F	39	T1N0M0	S	45-NA	—	FN	—

51		79/F	28	T2N0M0	S	19.3–2.72	—	FN	—

Ex: surgical excision; [^18^F]FDG-PET/CT: 2-deoxy-2-[^18^F]fluoro-D-glucose positron emission tomography/computed tomography; F: female; M: male; S: sporadic; Fam: familial; FN: false negative, TP: true positive; FP: false positive; MTC: medullary thyroid cancer; NA: not available.
